# Calcitriol-Mediated Hypercalcemia, Somatostatin Receptors Expression and 25-Hydroxyvitamin D_3_-1α- Hydroxylase in GIST Tumors

**DOI:** 10.3389/fendo.2021.812385

**Published:** 2022-01-26

**Authors:** Yiraldine Herrera-Martínez, María José Contreras González, Sergio Pedraza-Arévalo, Maria del Carmen Guerrero Martínez, Ángela Rodrigo Martínez, Alberto González Menchen, Maria Angeles Blanco Molina, Maria Angeles Gálvez-Moreno, Alberto L. Moreno-Vega, Raúl M. Luque, Aura D. Herrera-Martínez

**Affiliations:** ^1^ Maimonides Institute for Biomedical Research of Córdoba, Cordoba, Spain; ^2^ Nuclear Medicine Service, Virgen del Rocio University Hospital, Seville, Spain; ^3^ Medical Oncology Service, Reina Sofia University Hospital, Córdoba, Spain; ^4^ Department of Cell Biology, Physiology and Immunology, University of Cordoba, Cordoba, Spain; ^5^ Internal Medicine Service, Reina Sofia University Hospital, Córdoba, Spain; ^6^ Pathology Service, Reina Sofia University Hospital, Córdoba, Spain; ^7^ Endocrinology and Nutrition Service, Reina Sofia University Hospital, Córdoba, Spain; ^8^ CIBER Physiopathology of Obesity and Nutrition (CIBERobn), Cordoba, Spain

**Keywords:** hypercalcemia, calcitriol, GIST tumor, somatostatin receptors, corticoids

## Abstract

Hypercalcemia is a common complication in cancer patients Mainly caused by Parathyroid hormone-related protein (PTHrP) secretion and metastasis. Calcitriol secretion is a rare source of hypercalcemia in solid tumors, especially in gastrointestinal stromal tumors (GIST). We present a case report of a female patient with a 23 cm gastric GIST that expressed somatostatin-receptors and presented with severe hypercalcemia due to calcitriol secretion. Calcium control was achieved with medical treatment before the use of targeted-directed therapies. Surgery was performed and allowed complete tumor resection. Two years later, patient remains free of disease. Molecular analysis revealed the mRNA expression of 25-hydroxyvitamin D_3_-1-hydroxylase (1αOHase) and vitamin-D receptors in the tumor cells, confirming the calcitriol-mediated mechanism. Furthermore, the expression of the endotoxin recognition factors CD14 and TLR4 suggests an inflammatory mediated mechanism. Finally, the expression of somatostatin-receptors, especially *SST2* might have been related with clinical evolution and prognosis in this patient.

## Introduction

Humoral hypercalcemia of malignancy (HHM) is a common complication in cancer patients. Parathyroid hormone-related peptide (PTHrP) secretion is the most common cause, followed by metastatic bone disease (1, 2). Hypercalcemia due to 1,25-dihydroxyvitamin(OH)D_3_ [calcitriol;1,25(OH)_2_D_3_] overproduction is a rare cause of hypercalcemia (3). Specifically, sarcoidosis (49%), tuberculosis (8%) and lymphomas (17%) are the most frequent causes (4–7) of calcitriol-induced hypercalcemia; despite this, some other non-granulomatous diseases including solid tumors (5%) have been described (7). It is hypothesized that, in non-granulomatous diseases, macrophages and lymphocytes express 25-hydroxyvitamin D_3_-1-hydroxylase (1αOHase), which is responsible for conversion of 25-hydroxi vitamin D [25(OH)D_3_] to 1,25(OH)_2_D_3_, resulting in hypercalcemia due to increased calcium and phosphorus absorption from intestine and bone, but the exact pathophysiological mechanism is still not well understood (6, 8–11).

Very few cases of gastrointestinal stromal tumor (GIST) resulting in hypercalcemia have been reported. Among them, three have been related to calcitriol overproduction (3, 12–16). Calcitriol-mediated hypercalcemia seems to be associated with worse outcomes, suggesting that calcitriol might be a marker of high-grade tumors (17). Clinical management is challenging due to the limited information and the high associated mortality.

## Case Report

A 66-year old woman presented with weight loss, anorexia and abdominal pain. She was previously diagnosed with hypertension, dyslipidemia and subclinical hypothyroidism, which were treated and controlled. Blood analysis showed hypercalcemia (15.4 mg/dL); phosphorus (2.6 mg/dL), magnesium, kidney and liver function were normal. Parathormone (PTH) was slightly decreased (12.8 pg/mL, reference range (RR):14-72 pg/mL), PTH-related peptide was undetectable (<1.1 pmol/L), 25(OH)D_3_ was decreased (5 ng/dl,RR: 30-100 ng/dl) and calcitriol was elevated (119 pg/mL; RR:18-71 pg/mL).

**Figure 1:**
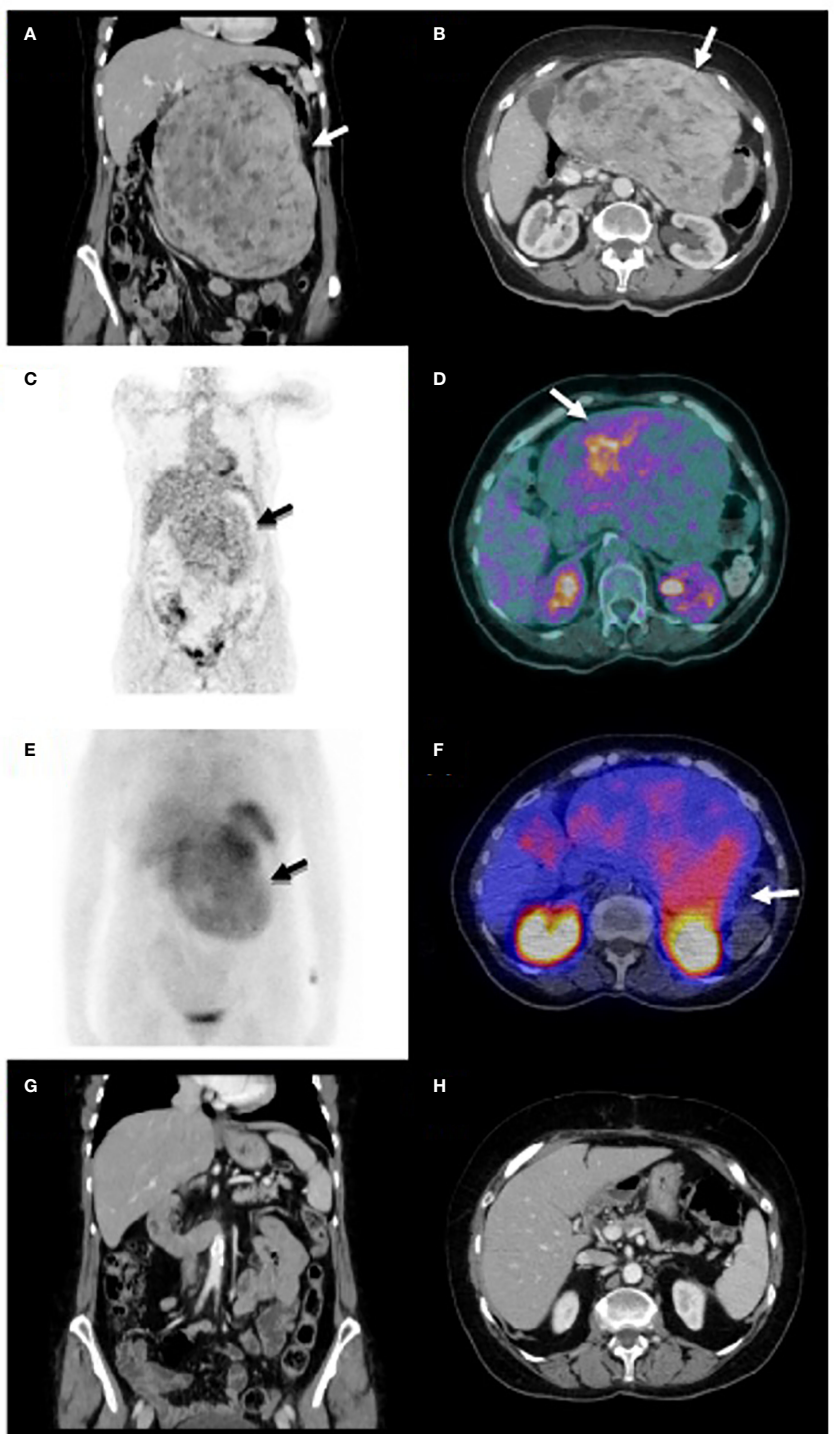
Coronal and axial CT views at diagnosis **(A, B)** show an abdominal solid mass (23x13x18 cm), with several necrotic-cystic zones, closely located to the lesser curvature of the stomach, which displaced the duodenum, pancreas, aorta and cave vein. Coronal and axial images of 18-FDG PET/CT scan **(C, D)** reveal increased metabolic activity in the whole mass. Coronal and axial images of somatostatin receptor scintigraphy SPECT/CT show increased expression of somatostatin receptors **(E, F)**. Coronal and axial images of CT after surgery show complete tumor resection **(G, H)**.

She was admitted for controlling hypercalcemia and to determine the underlying cause. CT showed a well-localized abdominal solid mass (23x13x18 cm), with necrotic-cystic zones, closely located to the lesser curvature of the stomach, and displacing the duodenum, pancreas, aorta and cave vein (**Figures 1A, B**). The 18-FDG PET/CT scan showed increased metabolic activity in the whole mass (**Figures 1C, D**). Additionally, a somatostatin receptor scintigraphy SPECT/CT revealed increased expression of somatostatin receptors (**Figures 1E, F**). The guided biopsy reported a low-grade (GIST with increased expression of CD34, DOG1 and CKIT.

**Figure 2:**
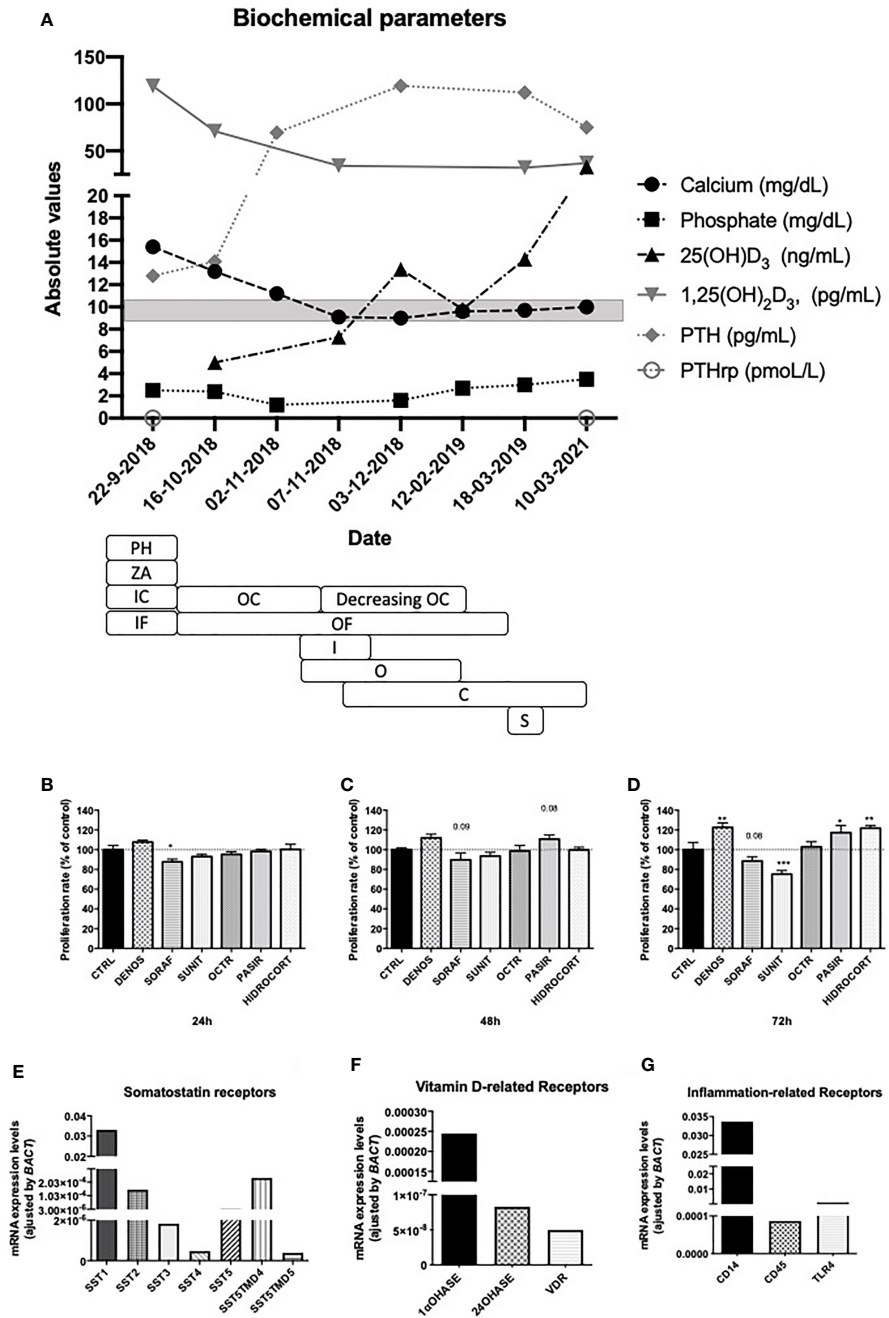
**(A)** Time-line representation of evaluated biochemical parameters since diagnosis until two years after surgery. Serum values of calcium, phosphate, PTH, PTHrP, 25(OH)D_3,_ 1,25(OH)_2_D_3_ are depicted. Medical treatment is also represented. PH, parenteral hydration; ZA, zolendronic acid; IC, intravenous corticoids; IV intravenous furosemide; OC; oral corticoids; OF, oral furosemide; I, imatinib; O, octreotide; C, calcifediol; S, surgery. The gray square represents normal calcium serum levels. Cell proliferation assay of primary cultures of the described GIST tumor after 24, 48 and 72 hours of incubation with denosumab, sorafenib, sunitinib, octreotide, pasireotide and hydrocortisone **(B–D)**. Molecular expression of somatostatin receptors **(E)**, vitamin D- related enzymes and receptors **(F)** and inflammation related genes **(G)** using qPCR. *p < 0.05; **p < 0.01; ***p < 0.001.

During admission, medical treatment with parenteral hydration, furosemide, intravenous corticoids and zoledronic acid was started. Patient was discharged with calcium levels of 13.2 mg/dL and close monitoring in the outpatient clinic. A single dose of denosumab 120 mg SC was administrated, and treatment with oral furosemide and corticoids (prednisone 1.5 mg/Kg body weight) was continued. Calcium was normalized after 6 weeks of treatment, and decreasing doses of corticoids and furosemide were started together with vitamin D supplementation. Imatinib was prescribed but administered during 6 weeks, since she presented with an atherothrombotic transient ischemic attack. Additionally, octreotide 10 mg IM was administered during three months. The evolution of serum calcium, calcitriol, other biochemical parameters and the respective treatment are depicted in **Figure 2A**. Surgery was performed five months after the diagnosis and total resection of the lesion was achieved (**Figure 1G, H**). According to the surgeon, tumor was smaller than expected according to the CT images. The pathology analysis revealed an 18-cm c-kit, CD34 and DOG1 positive GIST with necrosis and Ki67<1%. A mutation in exon 11 of the c-KIT gene was identified. Treatment was continued only with vitamin D. Fifty six months after diagnosis, the patient is asymptomatic, with normal serum calcium levels and no evidence of relapsed disease.

Serum calcium control was achieved in this patient before surgery, but the specific medical treatment and the underlying mechanism are unknown. According to the published cases, calcium control was not achieved in most of patients (18). Thus we aimed to evaluate the *in vitro* effects of the prescribed (or similar) drugs on cell proliferation, secretion and mRNA expression in a primary culture of the resected tumor. Specifically, hydrocortisone, denosumab, octreotide, pasireotide, sorafenib and sunitinib were evaluated. During the first 24h, sorafenib significantly decreased cell proliferation (**Figure 2B**), any significant change was observed after 48 hours (**Figure 2C**) but after 72h only sunitinib achieved this effect. Remarkably, denosumab, pasireotide and hydrocortisone increased cell proliferation at 72h (**Figure 2D**). Apoptosis and calcitriol secretion were undetectable (results not shown). The molecular analysis of tumor cells in the tumor sample reveled mRNA expression of somatostatin receptors (especially *SST1, SST2* and the truncated receptor *SST5TMD4*; **Figure 2E**). Additionally, the mRNA expression of the enzyme 1αOHase was confirmed in the tumor sample, even the expression of the enzyme 25-hydroxyvitaminD_3_24hydroxylase (24OHase), which hydroxylates 1,25(OH)_2_D_3_. mRNA levels of vitamin D receptors were also confirmed (**Figure 2F**). Furthermore, the expression of the inflammation related receptors CD14, CD45 and toll like receptor 4 (TLR4) were also confirmed (**Figure 2G**).

## Discussion

HHM affects approximately 20% of all cancer patients during their clinical course. In solid tumors, excessive secretion of PTHrP is the most common cause (80%). Structurally, PTHrP is similar to PTH in the first 13 amino acid sequences. In consequence, it binds the same receptor causing bone resorption, increased phosphate excretion and calcium reabsorption; but it does not have any effect on 1,25(OH)_2_D_3_ production (19). The second cause of HMM is bone metastases (20%), which release osteoclast activating factors, increasing bone-turnover and blood calcium levels. Commonly patients present with skeletal metastasis, low to low-normal PTH, PTHrP, and 1,25(OH)_2_D_3_ levels (19).

Other mechanisms, including calcitriol secretion, are uncommon. Specifically, ectopic secretion of calcitriol is the main mechanisms of hypercalcemia in granulomatous diseases and lymphomas (6, 8, 9). Calcitriol secretion by solid organ tumors is less frequent (7). Hypercalcemia in GIST tumors is also very uncommon, currently, only a few cases of GIST related hypercalcemia have been reported, only three of them related to calcitriol overproduction (3, 12–15).

In our case the two most common causes of HHM, PTHrP secretion and bone metastasis, were excluded by negative PTHrP values and absence of metastasis in imaging. Hyperparathyroidism was also ruled out by decreased level of PTH. No other findings in the PET-CT scan and the absence of monoclonal protein in serum and urine excluded other solid tumors and multiple myeloma. The only mechanism that could explain hypercalcemia in our case was increased production of calcitriol by tumor cells, since elevated serum levels of calcitriol were observed at initial presentation and progressively decreased in parallel to calcium concentrations. After calcium normalization, PTH serum levels raised and physiologically responded to 25(OH)D_3_ serum levels.

GISTs comprise 1% of gastrointestinal tumors, these mesenchymal neoplasms most commonly arise in the stomach and small intestine (16). The incidence ranges 10–15 per million per year, affecting both sexes equally with a median age of 60 years-old (20). In >90% of cases activating mutations in KIT gene are present, producing significant amounts of KIT transmembrane receptor tyrosine kinase (21). Clinical presentation and tumor characteristics perfectly match with our case report.

Despite the uncommon clinical presentation, the main matter of discussion in this case is calcium response after medical treatment. Clinical response to calcitonin, zolendronic acid and corticoids has been previously described (3); additionally, partial or total calcium control has been reported in patients treated with imatinib (3, 13). It is well-known that management of GIST improved since systemic therapy with tyrosine kinase inhibitors (TKI) was started, among them, imatinib is considered prototype drug (22, 23). In our patient, a single dose of this drug was administered, but calcium serum levels were already normalized; similar situation occurred with octreotide, which was administered based on the somatostatin receptor scintigraphy SPECT/CT results. One, or both of them could have been responsible for decreased tumor size; the *in vitro* experiments showed that only a TKI decreased tumor proliferation after 72 hours of incubation. Importantly, according to the cited cases reports, calcium control was achieved in one patient in parallel with tumor response, but in the other patient, calcium control was lost one-year after treatment due to tumor progression (3, 13).

Interestingly, our case expressed somatostatin receptors on imaging and in the molecular analysis. This expression has been previously described (24). As in our case, SST1 and SST2 represented the most expressed receptors. Furthermore, SST2 has been considered as a prognosis factor in GIST tumors, since decreased SST2 expression has been associated with decreased recurrence-free survival. Based on this, medical treatment with somatostatin analogs has been suggested as a therapeutic option in advanced tumors with receptors expression (24). Importantly, two years after surgery, our patient remains asymptomatic and disease-free.

Other authors have also evaluated the underlying mechanisms of HHM. Specifically, the expression of 1αOHase has been reported as responsible for HHM in dysgerminomas, and the expression of this enzyme in combination with the endotoxin recognition factors CD14 and TLR4 in tumor cells suggested that inflammatory mechanisms would have been responsible for hypercalcemia (25). The expression of 1αOHase, CD14 and TLR4 was also observed in our case, and might represent the putative mechanism of hypercalcemia, thus treatment with high-dose corticoids was probably responsible for calcitriol secretion control. CD45 levels in our case also reveal the inflammatory nature of the tumor.

In conclusion, hypercalcemia in GIST tumor patients is a rare phenomenon. To the best of our knowledge, this is a very rare case of calcitriol secretion with appropriate control calcium levels before TKI use. Additionally, our case is the first one that demonstrate tumor 1α-hydroxylase activity and vitamin D receptor expression, which could explain the increased levels of calcitriol and subsequent hypercalcemia.

## Ethics Statement

Informed consent has been obtained from the patient for publication of this case report and the accompanying images.

## Author Contributions

All authors have equally contributed with the preparation of this manuscript.

## Funding

This work was funded by Instituto de Salud Carlos III, co-funded by European Union (ISCIII- JR19/00050) and GETNE Junior Research Grant 2019.

## Conflict of Interest

The authors declare that the research was conducted in the absence of any commercial or financial relationships that could be construed as a potential conflict of interest.

## Publisher’s Note

All claims expressed in this article are solely those of the authors and do not necessarily represent those of their affiliated organizations, or those of the publisher, the editors and the reviewers. Any product that may be evaluated in this article, or claim that may be made by its manufacturer, is not guaranteed or endorsed by the publisher.

## References

[1] Wagner J Arora S. Oncologic Metabolic Emergencies. Emerg Med Clin North Am (2014) 32(3):509–25. doi: 10.1016/j.emc.2014.04.003 25060247

[2] Stewart AF. Clinical Practice. Hypercalcemia Associated Cancer N Engl J Med (2005) 352(4):373–9. doi: 10.1056/NEJMcp042806 15673803

[3] Hygum K Wulff CN Harslof T Boysen AK Rossen PB Langdahl BL. Hypercalcemia in Metastatic GIST Caused by Systemic Elevated Calcitriol: A Case Report and Review of the Literature. BMC Cancer (2015) 15:788. doi: 10.1186/s12885-015-1823-7 26499069 PMC4619287

[4] Baughman RP Teirstein AS Judson MA Rossman MD Yeager H Jr Bresnitz EA. Clinical Characteristics of Patients in a Case Control Study of Sarcoidosis. Am J Respir Crit Care Med (2001) 164(10 Pt 1):1885–9. doi: 10.1164/ajrccm.164.10.2104046 11734441

[5] Bell NH Shary J Shaw S Turner RT. Hypercalcemia Associated With Increased Circulating 1,25 Dihydroxyvitamin D in a Patient With Pulmonary Tuberculosis. Calcif Tissue Int (1985) 37(6):588–91. doi: 10.1007/BF02554911 3937578

[6] Seymour JF Gagel RF. Calcitriol: The Major Humoral Mediator of Hypercalcemia in Hodgkin’s Disease and non-Hodgkin’s Lymphomas. Blood (1993) 82(5):1383–94. doi: 10.1182/blood.V82.5.1383.1383 8364192

[7] Donovan PJ Sundac L Pretorius CJ d’Emden MC McLeod DS. Calcitriol-Mediated Hypercalcemia: Causes and Course in 101 Patients. J Clin Endocrinol Metab (2013) 98(10):4023–9. doi: 10.1210/jc.2013-2016 23979953

[8] Adams JS Gacad MA. Characterization of 1 Alpha-Hydroxylation of Vitamin D3 Sterols by Cultured Alveolar Macrophages From Patients With Sarcoidosis. J Exp Med (1985) 161(4):755–65. doi: 10.1084/jem.161.4.755 PMC2189055 3838552

[9] Cadranel J Garabedian M Milleron B Guillozo H Akoun G Hance AJ. 1,25(OH)2D2 Production by T Lymphocytes and Alveolar Macrophages Recovered by Lavage From Normocalcemic Patients With Tuberculosis. J Clin Invest (1990) 85(5):1588–93. doi: 10.1172/JCI114609 PMC296610 2159024

[10] Donovan PJ Achong N Griffin K Galligan J Pretorius CJ McLeod DS. PTHrP-Mediated Hypercalcemia: Causes and Survival in 138 Patients. J Clin Endocrinol Metab (2015) 100(5):2024–9. doi: 10.1210/jc.2014-4250 25719931

[11] Motlaghzadeh Y Bilezikian JP Sellmeyer DE. Rare Causes of Hypercalcemia: 2021 Update. J Clin Endocrinol Metab (2021) 106(11):3113–28. doi: 10.1210/clinem/dgab504 34240162

[12] George A. Metastatic Gastrointestinal Stromal Tumour Presenting as Hypercalcaemia–a Rare Occurrence. Clin Oncol (R Coll Radiol) (2008) 20(4):317–8. doi: 10.1016/j.clon.2008.02.002 18339524

[13] Jasti P Lakhani VT Woodworth A Dahir KM. Hypercalcemia Secondary to Gastrointestinal Stromal Tumors: Parathyroid Hormone-Related Protein Independent Mechanism? Endocr Pract (2013) 19(6):e158–62. doi: 10.4158/EP13102.CR 24013983

[14] Al-Moundhri MS Al-Thahli K Al-Kindy S Salam J Rao L. Metastatic Gastrointestinal Stromal Tumor and Hypercalcemia in a Patient With Ulcerative Colitis. Saudi Med J (2006) 27(10):1585–7. 17013488

[15] Beckers MM Slee PH. Hypercalcaemia in a Patient With a Gastrointestinal Stromal Tumour. Clin Endocrinol (Oxf) (2007) 66(1):148. doi: 10.1111/j.1365-2265.2006.02682.x 17201815

[16] Barbaryan A Bailuc S Poddutoori P Richardson A Mirrakhimov AE. Gastrointestinal Stromal Tumor Induced Hypercalcemia. Case Rep Oncol Med (2017) 2017:4972017. doi: 10.1155/2017/4972017 28484656 PMC5397646

[17] Shallis RM Rome RS Reagan JL. Mechanisms of Hypercalcemia in Non-Hodgkin Lymphoma and Associated Outcomes: A Retrospective Review. Clin Lymphoma Myeloma Leuk (2018) 18(2):e123–e9. doi: 10.1016/j.clml.2017.12.006 29361495

[18] Holick MF Schnoes HK DeLuca HF. Identification of 1,25-Dihydroxycholecalciferol, a Form of Vitamin D3 Metabolically Active in the Intestine. Proc Natl Acad Sci USA (1971) 68(4):803–4. doi: 10.1073/pnas.68.4.803 PMC389047 4323790

[19] Vakiti A Mewawalla P. Malignancy-Related Hypercalcemia. Treasure Island (FL: StatPearls (2021). 29494030

[20] Soreide K Sandvik OM Soreide JA Giljaca V Jureckova A Bulusu VR. Global Epidemiology of Gastrointestinal Stromal Tumours (GIST): A Systematic Review of Population-Based Cohort Studies. Cancer Epidemiol (2016) 40:39–46. doi: 10.1016/j.canep.2015.10.031 26618334

[21] Hirota S Isozaki K Moriyama Y Hashimoto K Nishida T Ishiguro S. Gain-Of-Function Mutations of C-Kit in Human Gastrointestinal Stromal Tumors. Science (1998) 279(5350):577–80. doi: 10.1126/science.279.5350.577 9438854

[22] Joensuu H Roberts PJ Sarlomo-Rikala M Andersson LC Tervahartiala P Tuveson D. Effect of the Tyrosine Kinase Inhibitor STI571 in a Patient With a Metastatic Gastrointestinal Stromal Tumor. N Engl J Med (2001) 344(14):1052–6. doi: 10.1056/NEJM200104053441404 11287975

[23] Otani Y Furukawa T Yoshida M Saikawa Y Wada N Ueda M. Operative Indications for Relatively Small (2-5 Cm) Gastrointestinal Stromal Tumor of the Stomach Based on Analysis of 60 Operated Cases. Surgery (2006) 139(4):484–92. doi: 10.1016/j.surg.2005.08.011 16627057

[24] Zhao WY Zhuang C Xu J Wang M Zhang ZZ Tu L. Somatostatin Receptors in Gastrointestinal Stromal Tumors: New Prognostic Biomarker and Potential Therapeutic Strategy. Am J Transl Res (2014) 6(6):831–40. PMC4297350 25628793

[25] Evans KN Taylor H Zehnder D Kilby MD Bulmer JN Shah F. Increased Expression of 25-Hydroxyvitamin D-1alpha-Hydroxylase in Dysgerminomas: A Novel Form of Humoral Hypercalcemia of Malignancy. Am J Pathol (2004) 165(3):807–13. doi: 10.1016/S0002-9440(10)63343-3 PMC1618616 15331405

